# The Native Egyptian as a Subject of Surgical Operation

**Published:** 1890-01

**Authors:** 


THE NATIVE EGYPTIAN AS A SUBJECT FOR SURGICAL OPERATION.—The native Egyptian is an extremely good subject for surgical operation. Clot Bey, the founder of modern medicine in Egypt, has it that "it requires as much surgery to kill one Egyptian as seven Europeans. In the native hospitals, the man whose thigh has been amputated at two o'clock is sitting up and lively at six." Shock is almost entirely unknown, and dread of an impending operation quite an exception. In explanation may be noted the resignation inculcated by their religion; the very small proportion of meat in, and the total absence of alcohol from their diet, and in general their regular, abstemious, out-of-door life.

